# Sudan Ebolavirus VP35-NP Crystal Structure Reveals a Potential Target for Pan-Filovirus Treatment

**DOI:** 10.1128/mBio.00734-19

**Published:** 2019-07-23

**Authors:** Sara Landeras-Bueno, Shun-ichiro Oda, Michael J. Norris, Zhe Li Salie, Javier Guenaga, Richard T. Wyatt, Erica Ollmann Saphire

**Affiliations:** a La Jolla Institute for Immunology, La Jolla, California, USA; b Department of Immunology and Microbiology, The Scripps Research Institute, La Jolla, California, USA; c Skaggs Institute for Chemical Biology, La Jolla, California, USA; Washington University School of Medicine; University of Pittsburgh School of Medicine

**Keywords:** RNA virus replication, Sudan virus, crystal structure, ebolavirus, nucleoprotein, phosphoprotein

## Abstract

The filoviruses are etiological agents of life-threatening hemorrhagic fever with high mortality rate and risk of potential outbreak. Among members of this family, the Ebola (EBOV), Sudan (SUDV), and Marburg (MARV) viruses are considered the most pathogenic for humans. The ebolavirus nucleoprotein (NP) is the most abundant protein in infected cells and is essential for viral transcription and replication; thus, it represents an attractive target for therapeutic intervention. Here, we present the structure of SUDV NP in complex with the amino-terminal portion of the phosphoprotein VP35 at 2.3 Å. This structure captures VP35 chaperoning SUDV NP in a monomeric and RNA-free state. This transient state has been proposed to be key to maintaining a pool of monomeric and RNA-free NPs prior to NP-NP polymerization and encapsidation of the viral RNA genome. This structure also reveals a newly visualized interaction between NP and VP35, a well-defined beta sheet that is not present in previous structures. Affinity binding assays demonstrate that this beta sheet is essential for maintaining the high-affinity interaction between VP35 and a hydrophobic pocket on SUDV NP, and electron microscopy indicates the importance of this binding interaction to the oligomeric state and assembly of NP in human cells. Complementary structure-directed mutagenesis identifies critical residues conserved across the filovirus family that could be targeted by broadly effective antivirals.

## Abstract (precis)

Outbreaks of the filoviruses can be unpredictable in timing, location, and identity of the causative virus, with each of Ebola virus, Sudan virus, Bundibugyo virus, and Marburg virus reemerging in the last several years to cause human disease with 30 to 90% lethality. The 2014–2016 outbreak in particular, with nearly 30,000 patients, highlighted the ability of these viruses to emerge unexpectedly and spread rapidly. Two ebolavirus outbreaks have emerged this year, yet we still lack FDA-approved drugs with pan-filovirus activity to treat existing and emergent ebolaviruses. For all filoviruses, the interaction between the nucleoprotein and the phosphoprotein is essential for the virus life cycle and is a potential target for therapeutic intervention. In this report, we describe the crystal structure of the SUDV nucleoprotein with the interacting domain of the viral phosphoprotein, and we identify residues critical for high-affinity interaction and for control of the oligomeric state of the nucleoprotein. Structural comparison of this heterodimer with other members of the filovirus family allowed us to find conserved and essential atomic features that will facilitate understanding of the virus life cycle and the rational design of antivirals.

## INTRODUCTION

The ebolaviruses belong to the family *Filoviridae* and cause hemorrhagic fever in humans with high case fatality (1). Six separate ebolaviruses are known: Ebola virus (EBOV), Sudan virus (SUDV), Bundibugyo virus (BDBV), Reston virus (RESTV), Taï Forest virus (TAFV), and the recently discovered Bombali virus (BOMV) (2). Outbreaks of each of these viruses are unpredictable in timing and location. The 2013–2016 epidemic occurred more than 1,000 miles from any previously known location of Ebola virus (http://apps.who.int), it spread to several continents (3), and it caused 11,323 reported deaths (http://www.who.int/csr/disease/ebola/situation-reports/archive/en/). Prior to this episode and the ongoing outbreak in the Democratic Republic of Congo, the largest ebolavirus outbreak was caused by Sudan virus, in the Gulu district of Uganda, where it infected 425 people with ∼50% case fatality (4). Sudan virus has caused six outbreaks among humans and is the second-most frequently emerging ebolavirus.

Ebolaviruses are filamentous enveloped viruses that contain a negative-sense RNA genome of ∼19 kb (1, 5–7). These single-stranded RNAs are covered by multiple copies of nucleoprotein (NP) (8) and are associated with an RNA-dependent RNA polymerase (L) (9, 10), a phosphoprotein polymerase cofactor (VP35) (11–16), and a transcription activator (VP30) (12, 17, 18). This ribonucleoprotein complex is known as the nucleocapsid (NC) and is responsible for transcription and replication of the viral genome (1, 19). When analyzed by electron microscopy, the NC appears as a supercoiled structure (20–22). The helical organization of the NC is primarily determined by the NP, as suggested by the observation that complexes of NP with cellular RNA adopt similar helical structures (20, 23). The formation of these macromolecular complexes results from two coupled and cooperative activities of NP: polymerization and RNA binding (21, 22). Polymerization of NP is facilitated by insertion of the N-terminal portion of each NP monomer, known as the oligomerization arm, into a binding site on the adjacent monomer. The C-terminal end of each NP likely undergoes a conformational change during polymerization, in which a C-terminal alpha helix extends to the vicinity of the RNA-binding domain, forming a positive-charge tunnel that encircles the RNA and promotes RNA encapsidation (21, 22).

In nonsegmented, negative-sense (NNS) RNA viruses, premature polymerization of the NP and nonspecific binding of cellular RNA is prevented by binding of the newly synthesized NP to the phosphoprotein (P). This interaction builds a complex designated N^0^-P (24–27). Formation of this N^0^-P complex has been proposed to be key to providing monomeric and RNA-free NPs as a substrate for encapsidation of the viral RNA genome and the formation of the NC (25). Within the filovirus family, two related N^0^-P structures have been solved for EBOV (28, 29) and MARV (30). In these crystal structures, the N-terminal domain of NP (NPcore), responsible for RNA encapsidation and NC condensation, was solved in complex with the chaperone domain of VP35. We term this complex VP35-NPcore.

Despite the high pathogenicity of SUDV, a detailed understanding of the structure of monomeric NP, its capacity for self-polymerization, and the mechanisms underlying these processes are still lacking. In the present study, we demonstrate that the N-terminal domain of SUDV VP35 chaperones SUDV-NPcore in a monomeric and RNA-free state. Purification of this heterodimer complex allowed us to report the crystal structure of SUDV VP35-NPcore to 2.3-Å resolution. The structure revealed the characteristic bi-lobed configuration expected of the filovirus NP with an RNA-binding cleft in between. The structure, however, shows important differences, such as additional sites of interaction, including assembly of a short three-stranded beta sheet between SUDV VP35 and NP that were absent from previous structures of EBOV (28, 29) and MARV (30). Complementary characterization of a panel of interaction mutants highlighted the most relevant substitutions for the SUDV NP-VP35 protein-protein interaction and suggest critical interacting residues that could guide the design of antivirals for broad protection against SUDV, EBOV, and MARV.

Our data also contribute to the understanding of SUDV NP polymerization. Structural comparison of the monomeric, RNA-free NP-VP35 structure with the polymerized NP contained in the native viral NC (21) revealed the existence of a conserved hydrophobic pocket homologous to that found in other members of the NNS virus family (26, 27). Our complementary electron microscopy images of SUDV NC-like structures purified from human cells suggest that the differential occupancy of this hydrophobic pocket is key for the modulation of the NP polymerization state during NC assembly. NP is maintained in a monomeric and RNA-free state as VP35 occupies this hydrophobic pocket. When VP35 is displaced by the N-terminal oligomerization arm from an adjacent NP, NP polymerizes and encapsidates RNA. These results provide a structural basis to support a common model of NC assembly across the *Filoviridae* family of chaperoning NP in a monomeric RNA-free state that is required for replication-coupled formation of the NC around newly synthesized viral RNAs. This new knowledge will help in the identification of universal targets for antiviral treatments against members of the *Filoviridae* family.

## RESULTS AND DISCUSSION

### The N-terminal domain of SUDV VP35 chaperones the SUDV NP.

**FIG 1:**
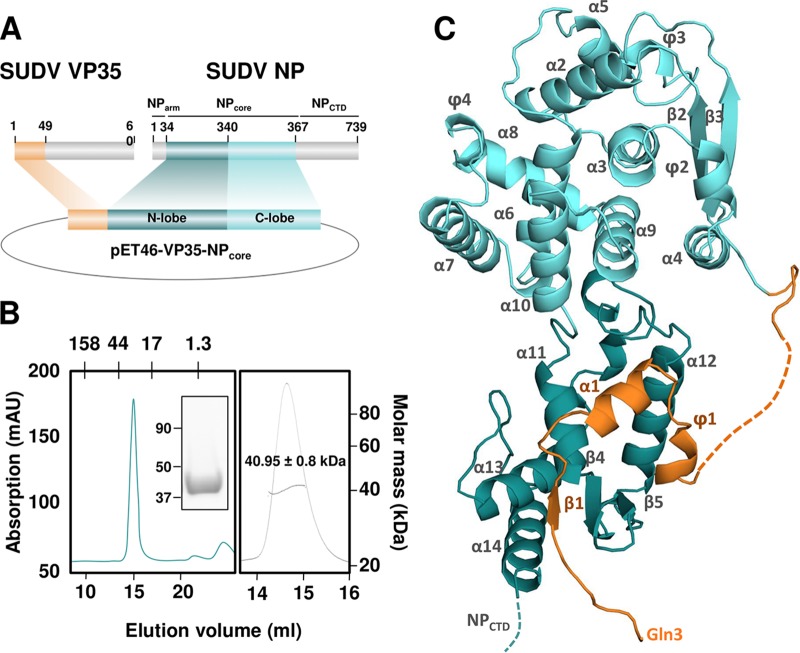
Crystal structure of the SUDV VP35-NPcore complex. (A) Schematic architecture of SUDV VP35 and NP proteins. NParm, N-terminal oligomerization arm of NP; N-lobe, NPcore (residues 34 to 367), N-terminal domain of NPcore; C-lobe, C-terminal domain of NPcore; NP_CTD_, C-terminal domain of NP; NPcore, N-lobe plus C-lobe. (B, left) Size exclusion chromatography of the SUDV VP35-NPcore complex. The molecular mass of standard protein markers (in kDa) is shown at the top. For comparison, the theoretical mass of the VP35-NPcore complex is 42.9 kDa. SDS-PAGE analysis of pure SUDV VP35-NPcore and the molecular mass of standard protein markers (in kDa) are shown inset. (Right) SEC-MALS analysis of SUDV VP35-NPcore showed a molecular mass of approximately 42 kDa. (C) Ribbon diagram of the 2.3-Å structure of SUDV VP35-NPcore. The N-terminal and C-terminal domains of NPcore are shown in cyan and dark cyan, respectively. VP35 visible from residue Gln3 is shown in orange. Missing (disordered) residues are presented by dotted lines.

The association of NP with VP35 has been shown to play a critical role during the encapsidation of viral RNA (28–30). To explore the structure and function relationships of SUDV NP and VP35, we determined their interactions at the atomic level by X-ray crystallography. The capacity of many NNS viruses to prevent premature NP polymerization via the N-terminal domain of the phosphoprotein prompted us to express and purify the chaperone domain of SUDV VP35(1-49) (28) fused to the SUDV NPcore region (residues 34 to 367) (Fig. 1A). This chaperone domain was identified previously by sequence homology with the Ebola virus chaperone domain and has proved sufficient to maintain NP in a monomeric state (28). The resulting fusion protein, termed SUDV VP35-NPcore, was expressed in Escherichia coli and purified by metal-affinity, ion-exchange, and size exclusion chromatography (Fig. 1B, left). The purified protein migrated as a single band of about 42 kDa on a denaturing 4 to 15% gradient PAGE (Fig. 1B, left) and was monomeric in solution (Fig. 1B, right). The sample did not contain RNA based on an *A*_260_/*A*_280_ ratio of 0.6. Together, these results verify that the first 49 amino acids of SUDV VP35 are sufficient to render the NP an RNA-free monomer in solution.

### Crystallization of a functional SUDV VP35-NPcore complex.

The SUDV VP35-NPcore complex was crystallized, and diffraction data were collected at 2.3 Å. The structure (Fig. 1C) was determined by molecular replacement using the structure of EBOV VP35-NPcore (PDB entry 4ZTG) as a search model. The electron density for most of the structure was clearly interpretable. Notably, this structure allowed, for the first time, visualization of the N-terminal region of VP35 beginning at the third residue. All residues of the SUDV VP35-NPcore complex were built into the final model, with the exception of one region for which density was not observed (residues V36 to A43 of VP35) (Fig. 1C). Data collection and model refinement statistics are summarized in Table 1.

**TABLE 1:** Data collection and refinement statistics

Parameter	SUDV VP35-NPcore^*a*^
Crystal	
Resolution range (last shell) (Å)	43.35–2.20 (2.28–2.20)
Space group	C121
Unit cell dimensions (Å)	*a* = 126.84, *b* = 33.75, *c* = 101.86; α = γ = 90°, β = 101.46°
No. of protein molecules (AU)	1
Solvent content (%)	59
Data collection	
Completeness (%)	99.53 (95.54)
No. of unique reflections	22,033
*I*/σ(*I*)	12.10 (0.90)
CC1/2^*b*^	0.99 (0.99)
Redundancy	6.80
Wilson B-factor (Å^2^)	54.54
Refinement	
*R* (%)/*R*_free_ (%)	21.10/24.80
RMSD bond length (Å)	0.003
RMSD bond angle (°)	0.56
B value for VP35 (Å^2^)	85.26
B value for NP (Å^2^)	74.75
No. of protein residues	334
Ramachandran analysis (%)	
Favored	98.07
Allowed	1.93
Outlier	0

a Statistics for the highest-resolution shell are shown in parentheses.

b CC1/2, Pearson correlation coefficient.

### The overall architecture of SUDV VP35-NPcore.

**FIG 2:**
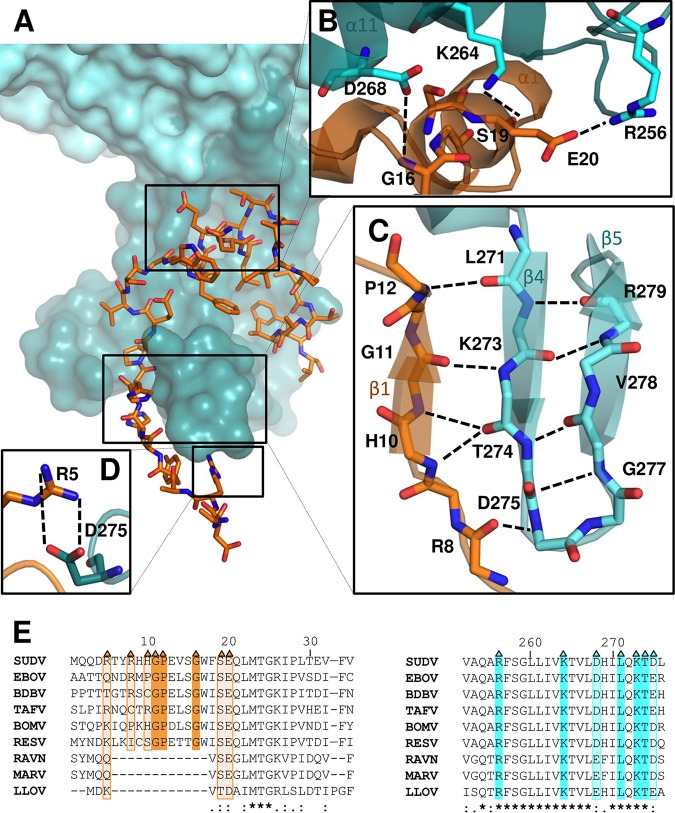
Protein-protein interactions between SUDV NPcore and its VP35 chaperone domain. (A) Surface representation of NP (in cyan) highlighting VP35 peptide (orange) interactions. (B to D) Interactions outlined by boxes in panel A. (B) R256, K264, and D268 in NP interact with E20, S19, and G16, respectively, in VP35. (C) A beta strand that was not visualized in previous VP35 structures complements a beta hairpin loop in NP. (D) R5 of VP35 forms a hydrogen bond with D275. Hydrogen bonds are represented by black dotted lines. (E) Sequence alignment of the VP35 chaperone domain (left) and NP (right). Triangles refer to the interacting residues shown in panels B to D. The interacting residues that are strictly conserved are highlighted in dark orange (for VP35) or dark cyan (for NP), and partially or nonconserved residues are highlighted in light orange (for VP35) or light cyan (for NP). Strictly conserved residues are indicated by stars, and partially conserved residues are indicated under the alignment by points according to ClustalW nomenclature.

The SUDV NPcore contains two major domains (Fig. 1A), an N-lobe (residues 34 to 340) and a C-lobe (residues 341 to 367), both of which are predominantly α-helical (Fig. 1C). Electrostatic analysis of the structure revealed that these lobes define a basic groove (see Fig. S1, lower, in the supplemental material) where RNA is bound in the native NC (Fig. S1, upper) (21, 22). The conformation and orientation of the VP35 chaperone domain are dictated by the interaction with NP. In this interaction, residues H10 to P12 of VP35 form a beta hairpin (β1), which is stabilized through main-chain hydrogen bonds by a longer beta sheet formed by β4 and β5 of NP. The medial part of VP35, residues S15 to L22, adopts an extended helical conformation (α1) located near the interface defined by the N- and C-lobes of NP. The protein-protein interface involves multiple hydrophobic contacts and nine hydrogen bonds (Fig. 2A to D). Comparison of NP and VP35 sequences across the *Filoviridae* family illustrated the substantial conservation of many of the interacting residues (Fig. 2A and E), suggesting the importance of this interaction for the virus life cycle.

10.1128/mBio.00734-19.1 FIG S1 Electrostatic surface potential of the nucleoprotein and location of its RNA binding region. (A and B) Electrostatic surface representation of polymerized EBOV NP interacting with RNA (PDB entry 5Z9W) (A) and monomeric SUDV NPcore bound to VP35 (PDB entry 6OAF) (B). The basic groove where the RNA is potentially bound is indicated by a black arrow. Proteins are colored by electrostatic potential as calculated with coulombic surface coloring in Chimera (45), where blue and red represent positive and negative regions, respectively. Both proteins are shown in two orientations, whereby the figure to the right shows the structure at a 90° rotation relative to the figure on the left. Download FIG S1, TIF file, 2.6 MB. Copyright © 2019 Landeras-Bueno et al. 2019 Landeras-Bueno et al. This content is distributed under the terms of the Creative Commons Attribution 4.0 International license.

### Unique features of SUDV VP35-NPcore structure.

**FIG 3:**
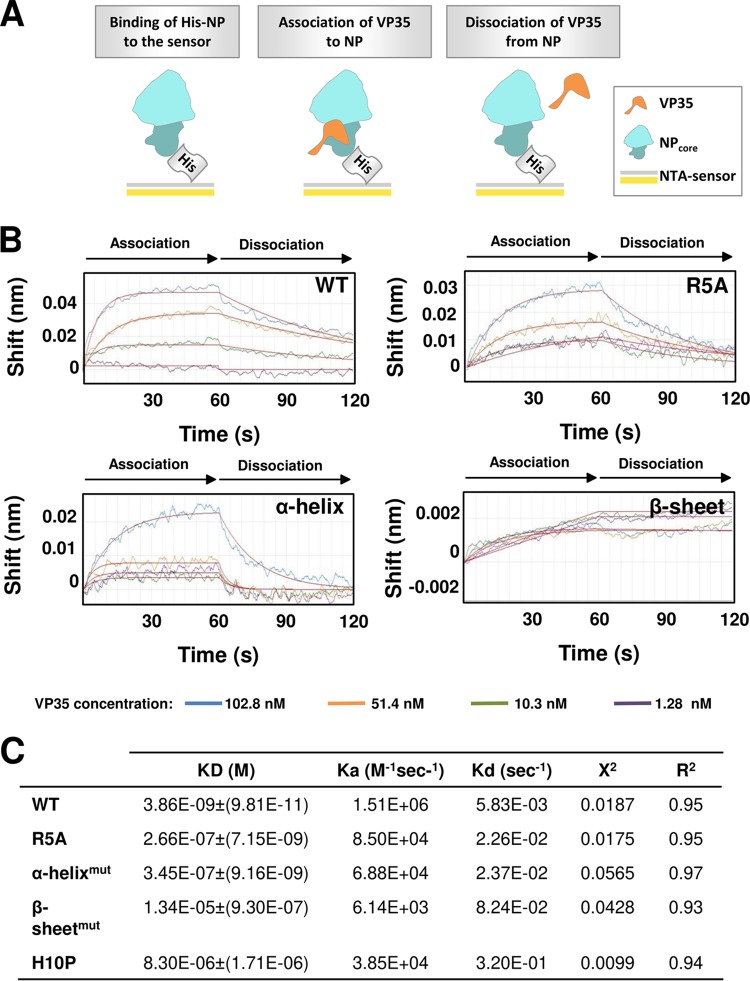
Mutational analysis of the interaction between SUDV NPcore and VP35 chaperone domain. (A) Schematic diagram of analysis by ForteBio Octet analysis. (B) Representative kinetic binding measurements between NP and different concentrations of VP35 wt or mutants that disrupt the hydrogen bond (R5A) or secondary structure (α-helix, β-sheet). Binding is expressed in nm and was monitored in real time using ForteBio Octet. Global fitting curves are shown in red. (C) Values of kinetic binding constants from a representative experiment (*n* = 3 independent biological replicates, 4 technical replicates of each). KD, affinity constant; Ka, association rate constant; Kd, dissociation rate constant. X^2^ is a measure of error between the experimental data and the fitted line. R^2^ indicates how well the fit and the experimental data correlate.

We superimposed the SUDV VP35-NPcore structure onto previously published structures of EBOV (PDB entry 4ZTG) and MARV (PDB entry 5F5O) NPcore bound to their corresponding VP35 chaperone peptides (Fig. S2). The overall structure of these fusion proteins is similar (Fig. S2A), facilitating the design of cross-species therapies that target conserved structural features. However, the comparison of these structures also revealed two previously unknown interactions between SUDV VP35 and NP: a hydrogen bond (VP35:R5-NP:D275) (Fig. 2D) and a structured beta strand (β1) that complements a beta loop in NP formed by β4 and β5 (Fig. 2C). This beta strand found in SUDV VP35 was not visualized in the previous structure of EBOV VP35 (Fig. S2B), so it is unknown if this interaction is conserved across the multiple ebolaviruses. In MARV, the corresponding residues form an alternative structure, an alpha helix (Fig. S2B). We used mutagenesis to explore the contribution of this beta strand assembly, together with the remainder of the identified interacting residues, to the binding of SUDV NP with VP35. We designed a series of mutants by replacing the amino acid residues involved in the three main interacting sites between SUDV VP35 and NP (Fig. 2B to D): salt-bridge interaction (single mutation VP35:R5A), beta strand (triple mutation VP35:H10G/G11P/P12G), and alpha helix (triple mutation VP35:G16P/S19A/E20A). Affinity measurements via biolayer interferometry binding analysis (Fig. 3A) showed that the SUDV VP35 wild-type (wt) chaperone domain bound SUDV NP with a dissociation constant (*K_D_*) of ∼3.86 × 10^−9^ M (Fig. 3B and C), in agreement with the affinity of other filoviruses (28–30). Binding of SUDV VP35:R5A to NPcore was 2 orders of magnitude lower (*K_D_* of ∼2.66 × 10^−7^ M) than the binding with the VP35 wt chaperone domain (Fig. 3B and C). Likewise, VP35 carrying mutations affecting the alpha helix interaction (VP35:G16P/S19A/E20A) showed a similar diminished affinity for NP (*K_D_* of ∼3.45 × 10^−7^ M) (Fig. 3B and C), indicating that both the hydrogen bond and alpha helix interaction have a significant effect on affinity. Importantly, replacement of the amino acid residues involved in the beta strand interaction (VP35:H10G/G11P/P12G) resulted in a dramatic decrease in the dissociation constant (*K_D_* of ∼1.34 × 10^−5^ M) (Fig. 3B and C). This result suggests that the newly visualized beta sheet interaction is essential for maintaining high-affinity binding between SUDV NP and VP35. Notably, two of the three residues involved in the beta strand formation, G11 and P12, are conserved across the filoviruses, as well as the amino acids in NP with which they interact (Lys273 and Leu271, respectively). The first residue (H10) is unique to SUDV VP35 (Fig. 2E). To characterize the unique contribution of SUDV H10 to the binding affinity, we mutated it to proline (VP35:H10P) to mimic the sequence of EBOV VP35. Surprisingly, this mutation resulted in a decrease in NP affinity by 3 orders of magnitude (*K_D_* of ∼8.30 × 10^−6^ M) (Fig. 3B), indicating that a nonproline residue at this site is key for binding and is responsible for the more ordered conformation of this region observed in SUDV. Only EBOV contains Pro10. All other known ebolaviruses (BDBV, RESTV, TAFV, and BOMV, in addition to SUDV) do not. Thus, the beta sheet observed here between VP35 and NP may be shared by BDBV, RESTV, TAFV, and BOMV, the non-EBOV ebolaviruses.

10.1128/mBio.00734-19.2 FIG S2 Superimposition of SUDV NPcore and its VP35 chaperone domain with the crystal structures of EBOV and MARV. (A) Structural alignment of the SUDV, EBOV (PDB entry 4ZTG), and MARV (PDB entry 5F5O) VP35 chaperone peptide fused with their corresponding NPcore, colored in cyan, pink, and green, respectively. (B) Main structural differences in VP35 chaperone peptide, expanded from the box outlined in panel A. Arrowhead 1 shows a more elongated alpha helix in MARV VP35 that is not present in EBOV and SUDV VP35, arrowhead 2 shows a well-defined beta sheet in SUDV VP35 that is not present in other structures, and arrowhead 3 shows a longer beta hairpin loop in SUDV NP that is one residue shorter in EBOV- and MARV-NP. Download FIG S2, TIF file, 2.6 MB. Copyright © 2019 Landeras-Bueno et al. 2019 Landeras-Bueno et al. This content is distributed under the terms of the Creative Commons Attribution 4.0 International license.

In conclusion, the residues involved in the beta sheet formation visualized in this structure are key for the binding between SUDV VP35 and NP and, hence, are key for chaperoning NP into its RNA-free monomeric state. The disruption of this key interaction could trigger the unregulated premature polymerization of NP, preventing the recruitment of this essential protein into the viral nucleocapsids and blocking the progression of the SUDV infection cycle.

### Full-length SUDV VP35 also chaperones SUDV NP in human cells.

**FIG 4:**
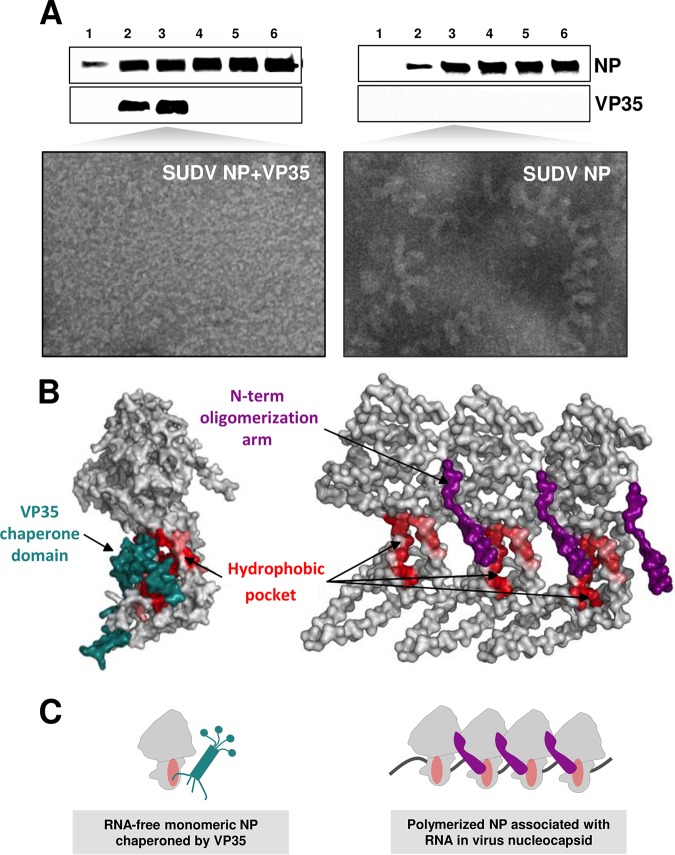
Full-length SUDV VP35 chaperones full-length SUDV NP in human cells. (A) Negative-stain EM images of monomeric SUDV NP (left) or SUDV NC-like structures (right) from HEK293T cells cotransfected with equal amounts of SUDV NP and VP35 tagged with HA (left) or SUDV NP with an equal amount of empty vector (right). Three days after transfection, the cells were lysed and NP was purified by ultracentrifugation in discontinuous CsCl gradients. A total of six fractions were collected, and the third fraction from the top was visualized by negative staining. Western blot using an anti-NP and anti-HA antibody of all fractions in the gradient is shown at the top. This experiment is representative of 3 independent experiments. (B) Comparison of monomeric SUDV NPcore bound to VP35 (left) with the polymerized EBOV NP present in native nucleocapsid structures (PDB entry 6C54) (right). VP35 (green) and the oligomerization arm of the adjacent NP (purple) occupy the same hydrophobic pocket (red) in NPcore. (C) Schematic representation of monomeric, RNA-free NP associated with VP35 (left) that could form the substrate for viral nucleocapsid assembly (right). The NP oligomerization arm (purple) must displace the VP35 chaperone domain (green) from the hydrophobic pocket of NP (red circle) in order to polymerize NP and bind viral RNA (gray line).

We next investigated whether full-length SUDV VP35 can similarly chaperone NP into a monomeric state in a system relevant to natural infection. Full-length SUDV NP was expressed alone or in the presence of full-length SUDV VP35 in HEK293T cells. After 3 days, total cell extracts were isolated and purified in discontinuous CsCl gradients by ultracentrifugation. The fraction showing the largest amount of VP35 enrichment or the corresponding one in the absence of VP35 was imaged by electron microscopy (EM) (Fig. 4A, lower). Expression of NP alone leads to NP polymerization, as evidenced by the presence of loose native-like structures (Fig. 4A, right) similar to previously described EBOV NC-like structures (20). In contrast, such NC-like structures were dramatically reduced in fractions in which NP was coexpressed with either full-length VP35 (Fig. 4A, left) or the VP35 chaperone domain alone (data not shown). These functional data are consistent with the chaperoning capacity of VP35 captured in the crystal structure.

The molecular mechanism by which VP35 inhibits NP polymerization was revealed for the homologous proteins of other NNS viruses (24–30). The chaperone domain of the phosphoproteins is proposed to mesh into a hydrophobic pocket on the NPcore, which, in the context of the nucleocapsid, is occupied by the oligomerization arm of an adjacent NP (Fig. 4B, right). We believe that competition for the NP hydrophobic pocket between the chaperone domain of VP35 and the NP oligomerization arm is what regulates NP polymerization: NP is monomeric when VP35 occupies the hydrophobic pocket and polymeric when the pocket is free. We illustrated the hydrophobic pocket in our SUDV NPcore structure by coloring the NP interacting residues, or those in the vicinity, by the Eisenberg hydrophobicity scale with a range of 1.380 to −2.530, most (red) to least (white) hydrophobic (Fig. 4B, left). The hydrophobicity of these residues is conserved among the filoviruses, supporting a common model of NC assembly (Fig. 4C).

Based on the evidence presented here and in previous studies (28–30), we propose the following sequence of events for the incorporation of NP into the NC in infected cells: newly synthesized SUDV NP is bound to SUDV VP35, which maintains NP in a monomeric and RNA-free state (Fig. 4A, left). VP35 thereby prevents the premature polymerization of NP in the absence of viral genomes and also prevents polymerization on cellular RNAs (24–30). At sites of viral RNA synthesis, VP35 releases NP, allowing it to polymerize with other copies of NP contained in the growing NC. Stepwise addition of NP promotes encapsidation of the viral RNA. The fact that coexpression of the VP35 chaperone domain and NP in human cells represses the formation of NC-like structures (Fig. 4A) suggests that the affinity of the chaperone domain of VP35 for NP is higher than that of the NP-NP interaction. Simultaneously, other viral proteins interact with VP35 and NP, orchestrating NC encapsidation. One main factor here is that VP40 interacts with the C-terminal domain of NP, increasing the compaction of the NC (20) and ensuring that VP35 cannot disrupt the NP-NP interaction in this conformation (28).

### NP-VP35 interface as a pan-antiviral target.

**FIG 5:**
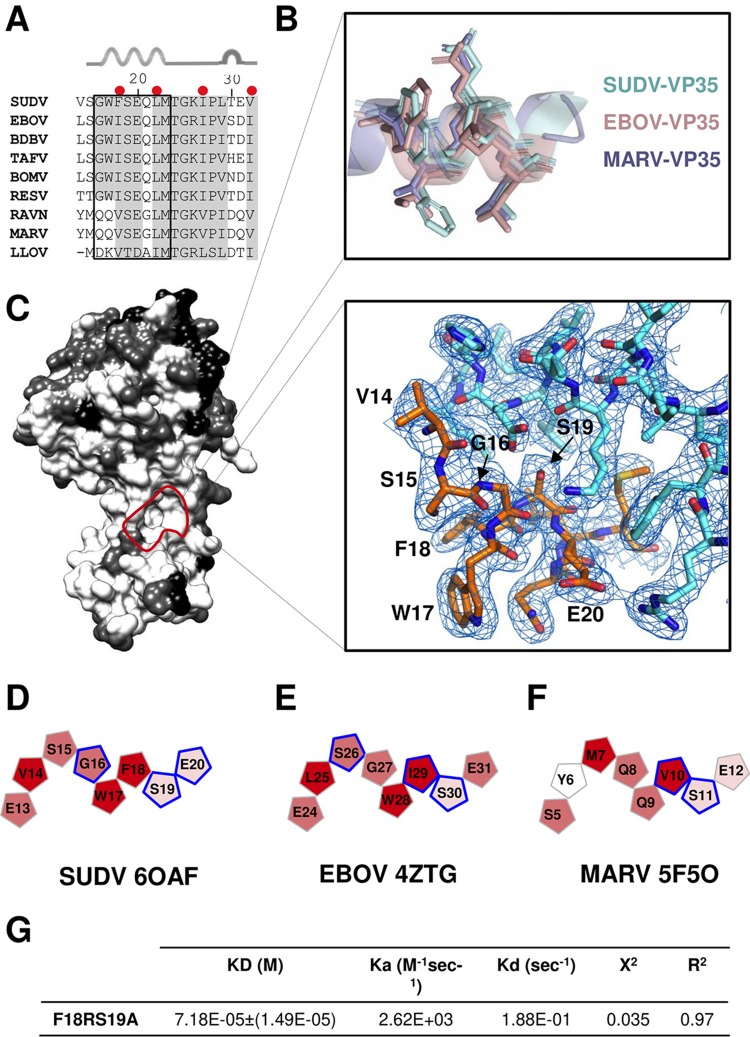
Hydrophobic binding surface between VP35 and NP could serve as a therapeutic target. (A) Multiple-sequence alignment of VP35. Conserved residues are shaded gray. Red dots above the alignment indicate the residues that contribute the most to the hydrophobicity of the chaperone domain. Secondary structure elements are indicated above the sequence. The alpha helix is represented by coiled ribbons and the T turn by a half circle. (B) Structural alignment of the conserved alpha helix of MARV (purple), EBOV (pink), and SUDV (cyan) VP35. (C) Protein surface conservation analysis of MARV (PDB entry 5F5O), EBOV (PDB entry 4ZTG), and SUDV VP35-NPcore. The conservation script in PyMOL was used to color residues according to the degree of conservation from conserved (white) to a more variable (black) residue. The conserved binding site in NP for the VP35 alpha helix shown in panel B is encircled by a pink dashed line. The interface of this interaction between SUDV NP and VP35 alpha helix and the corresponding map (2Fo-Fc, marine blue at 1.0 σ) are represented on the right. (D to F) Schematic representation of the most hydrophobic and conserved region of the VP35 chaperone domain of SUDV (D), EBOV (E), and MARV (F). Residues are colored by hydrophobicity scoring according to the Eisenberg scale with a range of 1.380 to −2.530, from most (red) to least (white) hydrophobic. Amino acids framed in blue interact with the NP of each filovirus and have been experimentally demonstrated to be important for this interaction. (G) Values of kinetic binding constants from a representative experiment (from three independent experiments) performed in a single run.

The presence of highly conserved residues (Fig. 5A to C) in the interacting interface between NP and VP35 of SUDV, EBOV, and MARV (Fig. 5D to F, respectively) could facilitate the development of small-molecule inhibitors that have pan-filovirus activity and, thus, could be used to treat a range of filovirus infections. Structural comparison of the NP-VP35 interface of these viruses revealed that the root mean square deviations (RMSD) ranged from 1.04 to 1.21 Å, with between 327 and 399 aligned residues and 55 to 80% sequence identity. Several structurally conserved patches are illustrated by coloring the residues of SUDV, EBOV, and MARV NP according to conservation (Fig. 5C). Among these is the conserved interaction site of the VP35 alpha helix 1 (Fig. 5C, dashed circle), a promising target for antiviral design. The amino acids forming the VP35 alpha helix (residues G16-M23) (Fig. 5A and B) are highly hydrophobic (Fig. 5D to F) and establish multiple interactions with NP. In particular, Ser19 forms a hydrogen bond to NP, the disruption of which affects VP35-NP binding affinity (28, 30). A Ser at this site is conserved among all filoviruses, save LLOV (Fig. 3E). Disruption of the hydrophobic residues in the vicinity of this Ser is also known to dramatically affect affinity of EBOV and MARV NP for their cognate VP35 (28, 30). We generated a double mutant (VP35:F18R/S19A) to determine the effect of the mutation of Ser19 to Ala together with the loss of hydrophobicity at position 18 on the SUDV NP interaction. Similar to observations for other filoviruses (28–30), the loss of hydrophobicity effectively abrogates binding (Fig. 5G). These results suggest that the residues of SUDV VP35:Phe18Ser19 and its homologs in other disease-causing filoviruses define a target for antiviral drug development. Disruption of this hydrophobic interaction between VP35 and NP could cause premature NP-NP polymerization and, hence, block filovirus replication.

The essential role of NP in viral RNA transcription and replication and the high conservation of NP across filoviruses makes antiviral therapies based on NP highly attractive (32–34). In fact, NP has been considered an ideal drug target for other NNS viruses, such as influenza A virus (35–40). In particular, it is known that the antiviral nucleozin, which blocks the polymerization of influenza NP, has potent effects *in vitro* and *in vivo* (36, 37). Since filoviruses are responsible for a high number of human casualties and have a potent emerging potential, definition of key, conserved interacting surfaces, essential for the virus life cycle, will be key to discovery of antiviral strategies.

## MATERIALS AND METHODS

### Recombinant protein expression and purification.

SUDV-NPcore(1-367) (strain *Boniface*), with an upstream hexahistidine tag followed by an enterokinase and tobacco etch virus (TEV) cleavage site, was cloned in the pET46 vector (Novagen). For coexpression of VP35 and NP, the VP35 (strain *Gulu*) chaperone domain [VP35(1-49)] was cloned in-frame upstream of NP(34-367) in the pET46 vector (Novagen). There was no linker between these two proteins. Plasmids were transformed in Rosetta2 pLysS Escherichia coli (Novagen) by heat shock, and starter cultures were grown with 18 μg/ml chloramphenicol and 100 μg/ml ampicillin in 50 ml Luria-Bertani broth (LB) overnight at 37°C and then used to inoculate 1-liter LB cultures containing 100 μg/ml ampicillin. Upon reaching an optical density at 600 nm (OD_600_) of 0.4 to 0.6, the cultures were induced with 0.5 mM isopropyl-β-d-thiogalactopyranoside and protein expression was allowed for 16 to 18 h at 25°C. The transformed cells were pelleted and lysed with a microfluidizer in 50 mM Tris-HCl, pH 8.0, 300 mM NaCl, 30 mM imidazole, and 2 mM β-mercaptoethanol (BME). Recombinant proteins were bound to nickel-nitrilotriacetic acid (Ni-NTA) beads for 1 h and eluted in 50 mM Tris-Cl, pH 8.0, 300 mM NaCl, 250 mM imidazole, and 2 mM BME. TEV protease (1 mg/ml; 0.5% [wt/wt]) was added to the Ni-NTA elutions to cleave the hexahistidine tags. The resulting sample was then dialyzed overnight in Snakeskin dialysis tubing, with 3,500-kDa pore size, in 50 mM Tris-Cl, pH 8.5, 100 mM NaCl, 5 mM BME. The protein was further purified by ion-exchange chromatography (Mono Q; GE Healthcare) using 25 mM Tris, 1 M NaCl, and 5 mM BME buffer for the gradient. The purified protein was concentrated using Amicon-3.5K filters and loaded onto a size exclusion column (Superdex 200; GE Healthcare). Protein concentrations were measured at 280 nm using a NanoDrop device (Thermo Scientific). The nucleic acid content was estimated by determining the *A*_260_/*A*_280_ ratio. Values of <0.7 were considered to be free of RNA.

### Size exclusion chromatography-multiangle light scattering (SEC-MALS).

Purified SUDV VP35-NP (200 μg) was applied to a size exclusion column (Superdex 200; GE Healthcare) in a buffer containing 25 mM Tris, pH 7.5, 0.15 M NaCl, and 0.02% NaN_3_ at a flow rate of 0.5 ml/min. Light-scattering data were collected on a Dawn MiniTreos (Wyatt Technologies) and analyzed with ASTRA (Wyatt Technologies).

### Crystallization and structure solution.

The VP35(1-49)–NP(34-367) fusion protein was crystallized at 20°C using a sitting-drop vapor diffusion technique by mixing an equal volume of protein solution (10.96 mg/ml) and reservoir solution (0.2 M potassium fluoride, pH 8.5, 0.1 M bicine, and 20% [wt/vol] polyethylene glycol 3350 [PEG3350]). Crystals were cryoprotected in 20.2% PEG200 prior to cryocooling in liquid nitrogen. Data used for the final structure were collected at the French National Synchrotron Facility (SOLEIL). Data sets were reduced with XDS (41), indexed in C121 (β = 101.46°), and merged with XSCALE (41). The crystal structure was solved by molecular replacement using PHASER in the CCP4 program suite (42). The Protein Data Bank (PDB entry 4ZTG) structure was used as a starting model. Solutions were rebuilt in Coot (43) and refined with Phenix (44).

### Purification of nucleocapsid-like structures.

HEK293T cells were transfected with 15 μg plasmid encoding SUDV NP alone or cotransfected with 15 μg hemagglutinin (HA)-SUDV VP35, or an empty plasmid, using TransIT-LT1 (Mirus). Three days after transfection, the cells were lysed with 10 mM Tris-HCl (pH 7.8), 0.15 M NaCl, 1 mM EDTA, and 0.1% Nonidet P-40, and NP was purified as previously described (20). Briefly, the clarified cell extract was added to a discontinuous CsCl gradient (25 to 40%) and centrifuged at 250,000 × *g* at 20°C for 1 h. Six gradient fractions were collected and analyzed by Western blotting to confirm the presence of NP and VP35, and the third fraction from the top of the gradient was analyzed by negative staining.

### Negative-stain electron microscopy.

Copper grids (carbon-coated, 400 mesh; Electron Microscopy Sciences) were glow discharged (75% argon–25% oxygen atmosphere, 0.02 W for 45 s) using a NanoClean model 1070 (Fischione). All samples were diluted to ∼1.5 mg/ml prior to staining. Initially, 3 μl of sample was applied to the grid. After 1 min, the sample was side-blotted manually with filter paper (VWR), and the grid was then stained with 2% uranyl acetate and side-blotted again. Specimens were examined on a Philips CM100 electron microscope (FEI) at 80 keV.

### Biolayer interferometry binding analysis.

His-SUDV-NP(34-367) was diluted to 3 μg/ml in Dulbecco’s phosphate-buffered saline (Gibco), 0.1% bovine serum albumin, 0.02% Tween and immobilized on NTA capture sensors (ForteBio) previously hydrated in the same buffer. SUDV VP35 peptides corresponding to residues 1 to 34 were synthesized by Biomatik and reconstituted at 2 mg/ml in dimethyl sulfoxide. The NP‐VP35 associations were performed using a 2-fold serial dilution of VP35 wild type or mutant (1.28 to 102.8 nM) for 60 s, and the dissociations were performed for 60 s. Affinity was determined via the kinetic constant once the buffer-subtracted Octet data were globally fit to a simple 1:1 model. All binding measurements were carried out on an Octet Red instrument (ForteBio), and results were processed using Octet software 10.0.1.

### Data availability.

The crystal structure was deposited in PDB under the accession number 6OAF.
