# Supplementary figures and images for: Sudan Ebolavirus VP35-NP Crystal Structure Reveals a Potential Target for Pan-Filovirus Treatment
Source: mBio. 2019 Jul 23;10(4):e00734-19. doi: 10.1128/mBio.00734-19 (PMC6650547; doi:10.1128/mBio.00734-19)

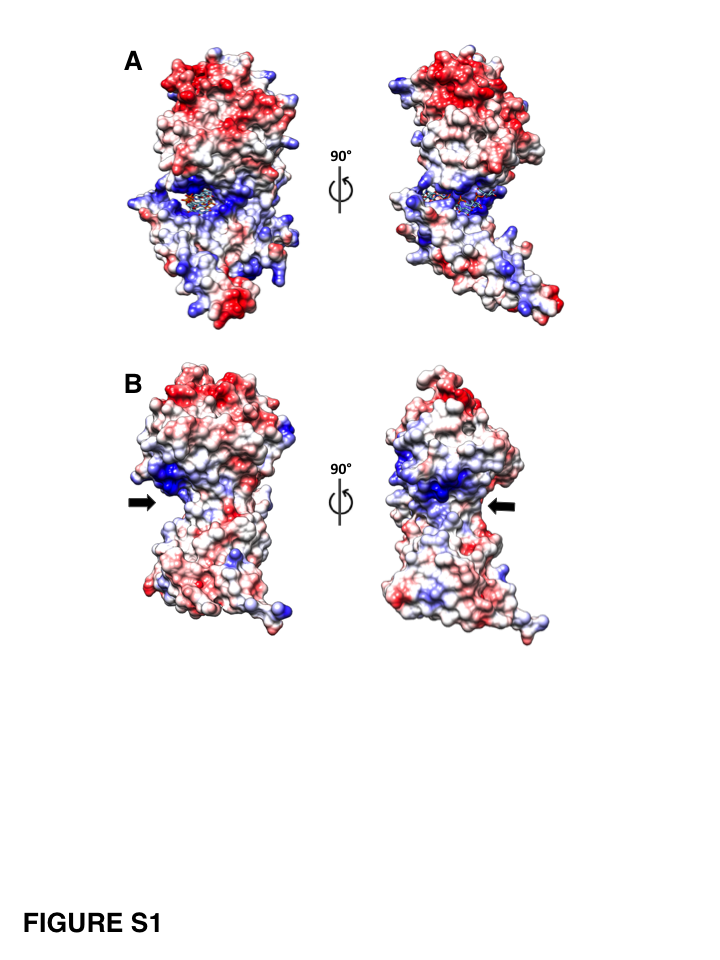

Supplement: FIG S1 [file mBio.00734-19-sf001.tif]

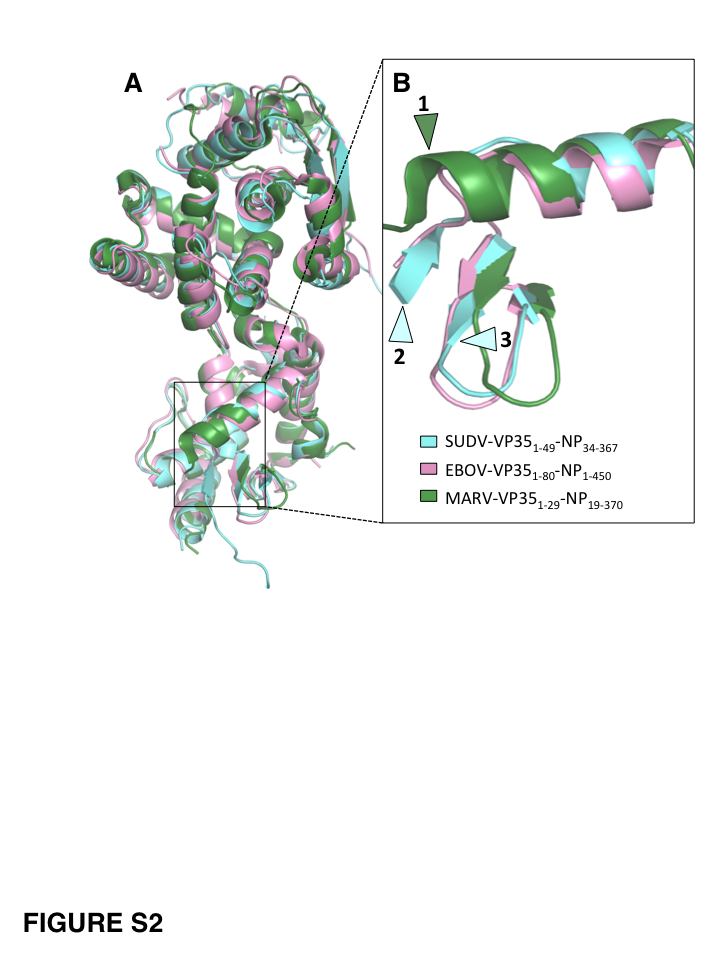

Supplement: FIG S2 [file mBio.00734-19-sf002.tif]
